# Role of GPs in shared decision making with patients about palliative cancer treatment: a qualitative study in the Netherlands

**DOI:** 10.3399/BJGP.2021.0446

**Published:** 2022-02-08

**Authors:** Danique W Bos-van den Hoek, Naomi CA van der Velden, Rozemarijn Huisman, Hanneke WM van Laarhoven, Dorien Tange, Jan Wind, Ellen MA Smets, Inge Henselmans

**Affiliations:** Department of Medical Psychology, Amsterdam UMC, University of Amsterdam; Amsterdam Public Health Research Institute; Cancer Center Amsterdam, Amsterdam.; Department of Medical Psychology, Amsterdam UMC, University of Amsterdam; Amsterdam Public Health Research Institute; Cancer Center Amsterdam, Amsterdam.; Department of Medical Psychology, Amsterdam UMC, University of Amsterdam, Amsterdam.; Cancer Center Amsterdam; Department of Medical Oncology, Amsterdam UMC, University of Amsterdam, Amsterdam.; NFK, Dutch Federation of Cancer Patient Organisations, Utrecht.; Department of General Practice, Amsterdam UMC, University of Amsterdam, Amsterdam.; Department of Medical Psychology, Amsterdam UMC, University of Amsterdam; Amsterdam Public Health Research Institute; Cancer Center Amsterdam, Amsterdam.; GP Training Institute, Department of Medical Psychology, Amsterdam UMC, University of Amsterdam; Amsterdam Public Health Research Institute; Cancer Center Amsterdam; Department of General Practice, Amsterdam UMC, University of Amsterdam, Amsterdam.

**Keywords:** cancer, general practice, hospital treatment, qualitative interview study, decision making, shared

## Abstract

**Background:**

GPs are well placed to enhance shared decision making (SDM) about treatment for patients with advanced cancer. However, to date, little is known about GPs’ views about their contribution to SDM.

**Aim:**

To explore GPs’ perspectives on their role in SDM about palliative cancer treatment and the requirements they report to fulfil this role.

**Design and setting:**

Qualitative interview study among Dutch GPs.

**Method:**

GPs were sampled purposefully and conveniently. In-depth, semi-structured interviews were conducted, recorded, and transcribed verbatim. Transcripts were analysed by thematic analysis.

**Results:**

Fifteen GPs took part in this study. Most of them reported practices that potentially support SDM: checking the quality of a decision, complementing SDM, and enabling SDM. Even though most of the GPs believed that decision making about systemic cancer treatment is primarily the oncologist’s responsibility, they did recognise their added value in the SDM process because of their gatekeeper position, the additional opportunity they offer patients to discuss treatment decisions, and their knowledge and experience as primary healthcare providers at the end of life. Requirements for them to support the SDM process were described as: good collaboration with oncologists; sufficient information about the disease and its treatment; time to engage in conversations about treatment; a trusting relationship with patients; and patient-centred communication.

**Conclusion:**

GPs may support SDM by checking the quality of a decision and by complementing and enabling the SDM process to reach high-quality decisions. This conceptualisation of the GP’s supporting role in SDM may help us to understand how SDM is carried out through interprofessional collaboration and provide tools for how to adopt a role in the interprofessional SDM process.

## INTRODUCTION

Patients with advanced cancer often deal with complex treatment decisions that depend on their values and preferences and, hence, require shared decision making (SDM).^1^^–^3 SDM is the process of decision making in which the healthcare professional and patient jointly discuss the pros and cons of different treatment options, as well as the patient’s values and preferences, to come to an agreed treatment decision.^3^^–^5 The relevance of SDM is underscored by the ethical considerations of patient-centred care and patient autonomy^6^ as well as by its positive impact on patient outcomes.^7^^–^13 In the context of palliative cancer care, most patients wish to be involved in making decisions about treatment.^14^^–^16 However, SDM is not always visible in observational studies. These studies suggest insufficient discussion of patients’ values and the option to refrain from disease-targeted treatment.^17^^–^20

It is increasingly recognised that SDM often takes place across multiple encounters with and between different clinicians.^21^^,^^22^ Although oncologists have expert knowledge about cancer treatment and often make the final choices about treatment with patients, GPs are well placed to enhance SDM and contribute to high-quality decisions.^23^ GPs have continuous relationships with patients, which can help them understand the medical and psychosocial context.^24^^–^26 They are accustomed to using a holistic approach to health problems and, generally, receive training in effective communication.^24^ A recent survey among patients with cancer found that the majority appreciate the GP’s involvement in cancer care after diagnosis.^27^ GPs’ involvement might also increase patient satisfaction with the decision,^28^ and patient satisfaction with GPs’ involvement,^29^ and may reduce decisional conflict for patients with advanced cancer.^30^

While GPs are involved in cancer screening, diagnosis, follow-up, and terminal palliative care, they seem to hardly be involved in decision making about cancer treatment.^25^^,^^29^^,^^31^^–^34 Despite suggestions that GPs should collaborate with oncologists to discuss treatment decisions with patients throughout the palliative phase,^35^^–^37 little is known about how GPs could contribute to SDM about advanced cancer treatment. By examining GPs’ existing practices in SDM about advanced cancer treatment from their own perspective and conceptualising them, the study wished to identify ways of strengthening GPs’ contribution and ultimately guarantee patient-centred care for people with advanced cancer. Thus, the aim was to explore GPs’ perspectives on their role in SDM about palliative cancer treatment and the requirements to fulfil this role.

**Table:** How this fits in

Shared decision making (SDM) is essential for patients with advanced cancer to ensure that their care matches their values and preferences. This study shows that GPs fulfil a supporting role in such treatment decision-making by checking the quality of the decision and by complementing or enabling SDM. This conceptualisation of the GP’s supporting role in SDM indicates how SDM is — or could be — carried out through relationship-based care and interprofessional collaboration, and exposes the complexities in interprofessional boundaries. Increased insight into and awareness of GPs’ contribution to the decision-making process may make their involvement more conscious and hence more effective.

## METHOD

### Design

Semi-structured, in-depth interviews were conducted with GPs. Data were analysed thematically. This report meets the standards for reporting qualitative research items.^38^

### Recruitment

GPs were eligible to participate in the study if they reported experience with patients with advanced cancer. GPs were recruited using purposeful and convenience sampling. The authors aimed to recruit a diverse sample of GPs with respect to sex, work experience, patient population, location (urban/suburban/rural), and type of practice (solo/duo/group practice). Interested GPs were sent information and an informed consent form.

### Data collection

Interviews were conducted face-to-face by two researchers in GPs’ consultation rooms. The researchers’ different backgrounds combined a conceptual approach to health care with practical experience in medicine, which helped them refine the interview guide and understand the experiences of GPs.

An interview guide was created and piloted with two GPs, resulting in small modifications (Box 1). The interview started with the participant reading the example case in Box 1 to set the scene to discuss the interview topics. The example case described a patient diagnosed with advanced stomach cancer who was considering palliative chemotherapy with a median survival gain of 5 months. While discussing the GP’s role in the example case the interviewer probed for general reflections and opinions on the following topics: the current and desired role of GPs in (conversations about) treatment decision making, as well as the requirements to be able to fulfil this role. The interviews lasted 30–45 minutes each and were conducted between October 2018 and January 2019. All participants signed informed consent forms and reimbursement was offered to all GPs for their time.

**Box 1.** Interview guide

**A. Short introduction to the interview** Introducing interviewer and research Explaining confidentiality and anonymity Signing informed consent Asking permission for audiorecording
**B. Substantive part of the interview** Presentation of a case study of a patient with incurable cancer who had to decide on treatment with a life-prolonging intent: *‘Pieter de Vries, aged 74, is single, has two daughters and one grandson. He lives on a remote farm. His wife died a few years ago. He has been dizzy for some time and has little appetite. He has also lost a lot of weight. After two visits to the GP, he was referred to the hospital and received bad news last week. He has stomach cancer, with metastases to the bones. The same week he had a conversation with the medical oncologist about treatment. He is eligible for palliative chemotherapy (CapOx). The median survival without chemotherapy is 6 months; with chemotherapy it is 11 months. Chemotherapy has side effects, including nausea or vomiting, fatigue, diarrhoea, tingling or numbness of the fingers and feet, and hand–foot syndrome (redness, chapping).’* Current role and ideal role for involvement in treatment decision making: — Current and desired role — Position with respect to other health professionals — Goals in conversations with patients — Steps or actions to reach these goals — Involvement in four steps of SDM: 1) informing about decision; 2) explaining options with pros and cons; 3) discussing preferences and supporting deliberation; and 4) making decision^5^ — Added value of conversation with GP — Moments for conversation Stimulating and restraining factors for fulfilling the role Needs required in order to fulfil the role
**C. Conclusion of the interview** Issues that were not addressed

### Data analysis

Interviews were audiorecorded, transcribed verbatim, anonymised, and analysed by thematic analysis.^39^ Coding was performed using MAXQDA software (versions 2018 and 2020). The approach was largely inductive; no coding sheet was prepared beforehand. The final categorisation of some themes and subthemes was informed, and likely influenced, by the simultaneous analysis of interviews with hospital nurses about their role in SDM about palliative treatment.^40^ Three researchers were involved in the coding process. Four interviews were double-coded independently by two researchers and discussed until they reached consensus. Another combination of two researchers repeated this for another four interviews. As coding agreement was high, one of these two researchers coded the consecutive seven transcripts, and they both discussed uncertainties until they reached consensus. During analysis, sections that referred to decision making in settings other than cancer care in the early palliative phase were not coded. Data saturation was monitored and considered achieved when no new substantial codes appeared in the final four interviews. A structure of categories and subcategories was developed throughout the analysis (Table 1). Two researchers refined potential overarching themes and the content of these themes was analysed to generate clear definitions and names for each theme. Participants received a short summary of the analysis to which they could respond. This is known as member checking, a technique for responder validation. Twelve GPs responded, and their comments led to small refinements.

**Table 1.** Themes and subthemes resulting from the thematic analysis

**Theme**	**Subthemes**	
**Involvement of GPs in the SDM process**	Moments to engage in conversations about treatment	
	Initiative for the GP–patient conversation	

**Supporting role of GPs in the SDM process**	Checking the quality of a decision (high-quality decision: conscious, informed, and appropriate)	Checking choice awareness Checking if decision isinformed Checking if decision is aligned with patient’s values
	Complementing SDM (adding to the decision-making process to reach a high-quality decision)	Increasing choice awareness Clarifying and adding information Exploring values and supporting preference construction
	Enabling SDM (organising activities to ensure reaching a high-quality decision)	Acting as a patient advocate Preparing upcoming conversations with the oncologist

**Interprofessional SDM: GPs' added value**	The unique position of GP in the healthcare system	
	Additional and different conversations about treatment	
	Primary healthcare provider in the terminal stage	

**Requirements for fulfilling a role in the SDM process**	Collaboration with the oncologist	
	Information about cancer and treatment options	
	Time to engage in conversations about treatment	
	Trusting relationship with patient	
	Patient-centred communication	

*SDM = shared decision making.*

## RESULTS

Fifteen Dutch GPs participated; they were based at 14 different practices representing 11 different health centres located in two provinces around Amsterdam. Eleven participants were recruited through the researchers’ network, one through snowballing, and three GPs responded to an invitation sent by the academic network of GPs of the authors’ institute. Table 2 gives the participants’ characteristics.

**Table 2.** Participant characteristics (*N* = 15)

**Variable**	***n***
**Years of experience, mean (range)^a^**	17.4 (4 to 30)
<10	4
10–20	4
>20	7

**Sex**	
Male	6
Female	9

**Patient population**	
Origin:	
Mixed	11
Mostly native-born	2
Mostly foreign-born	2
Age group:	
Younger than average	5
Average	5
Older than average	5

**Type of practice^b^**	
Type:	
Solo	3
Duo	8
Group	4
Location:	
Rural	2
Suburban	8
Urban	5

**Affinity with palliative care^c^**	
High	8
Average	6
Low	1

a *Mean number of years of experience of the study participants.*

b *GPs worked in 14 different practices; two GPs worked at the same practice.*

c *Combined score of received training on palliative care (yes/no) and indicated affection with palliative care (yes/no); indicating both was scored as high affinity, indicating either one was scored as average affinity, and indicating none was scored as low affinity with palliative care.*

The themes and subthemes resulting from the thematic analysis are outlined in Table 1.

### Involvement of GPs in the SDM process

#### Moments to engage in conversations about treatment

Most GPs mentioned having conversations with patients about their physical and psychological wellbeing on several occasions throughout the cancer trajectory: before referral and after patients received their diagnosis or other bad news, such as disease progression. These latter conversations were mentioned as possible starting points for GPs’ involvement in the SDM process:

*‘Often, when someone has received bad news, I’m definitely involved. So I get in touch with them and tell them I’d love to drop by and talk to you about this … to hear what you’ve learned. And whether you’ve decided for yourself yet?’*

(GP10)

#### Initiative for the GP–patient conversation

GPs differed in their opinions about whether patients, oncologists, or GPs should initiate such conversations. A major consideration was the importance of tailoring contact to patients’ needs, with some GPs waiting for patients to take the initiative while others contacted patients more proactively. Some GPs mentioned that, during cancer treatment, patients generally did not express needing GP involvement. Occasionally, oncologists actively referred patients to GPs to discuss treatment options:

*‘Only in rare cases, the oncologist goes: “talk to your GP about this”. Then it’s usually in the letter, uhm, and that’s of course fine by me. And that’s generally to do with me knowing the circumstances just that bit better.’*

(GP01)

### Supporting role of GPs in the SDM process

**Figure 1.**
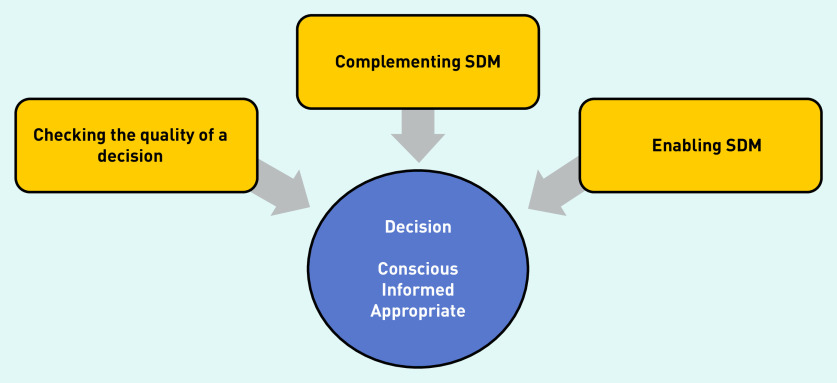
*Three strategies GPs use to support shared decision making (SDM).*

All GPs reported practices that potentially support SDM. These were organised into three categories: checking the quality of a decision, complementing SDM, and enabling SDM. GPs appear to deploy these strategies to ensure that decision making about treatments is conscious, where the patient is aware of the choice; well informed so the patient knows about the various possibilities and their pros and cons; and appropriate, in that the decision aligns with the patient’s values and preferences.^41^^,^^42^ **Figure 1** represents these strategies that GPs may use to reach high-quality decisions.

#### Checking the quality of a decision

GPs may check the quality of a decision by asking questions to check if there are any doubts or deficiencies for making high-quality decisions. GPs mentioned practices to check patients’ choice awareness, information level, and/or values and treatment preferences. For example, GPs reported how they queried the patient–oncologist decision-making conversation, tried to gauge patients’ understanding about treatment information, and probed for patients’ thoughts about and expectations regarding the proposed treatment:

*‘First, I check, like, “what have you been told? What stuck?” I ask them, “what have you heard from the specialist and what did you understand and can you tell me that in your own words?” Sometimes there’s a discrepancy already there.’*

(GP05)

*‘And then with a patient as in this* [example] *case, of course for myself I want to know a little bit more about, well, “how do you feel about this treatment, have you got any doubts, what would be important to you in the near future?”’*

(GP11)

#### Complementing SDM

This category comprised practices to add to the SDM process by, for example, introducing the choice, clarifying information, and supporting preference construction. With regard to increasing patients’ choice awareness, GPs mentioned how they explained that a choice needed to be made between different treatment options:

*‘And then I also like to say, as a GP: “ok, that may seem like the only option to you, but another option is actually to not do the chemo. Are you aware of that, that that is also an option? To say no?”’*

(GP10)

Additionally, GPs structured, clarified, and added information when they noticed that patients missed or misinterpreted information provided by the oncologist:

*‘When I notice any doubts, then I’ll definitely try to present as honest as possible a picture* [of the consequences of the treatment] *and explain that no treatment is also an option. And that it doesn’t mean they are on their own and that their life will end in suffering.’*

(GP10)

GPs sometimes supported patients’ preference construction by exploring their values, appraisals of treatment options, and, based on that, their preferences for treatment:

*‘One could look more at the bigger picture, like: “gosh, what is the meaning of life for you? What is quality of life for you? What do you expect from palliative chemotherapy? What do you expect to happen if you don’t get it?”’*

(GP02)

#### Enabling SDM

GPs were found to enable SDM by organising additional activities to ensure that the SDM process will continue beyond GPs’ direct involvement. GPs reported how they acted as an intermediary between the patient and oncologist, aided contact between the patient and oncologist, or helped prepare these conversations:

*‘I have called the oncologist once or twice with, “listen, you propose this, but I’m worried. This really is a very vulnerable person, we really shouldn’t do this”. And to have the specialist say: “that’s great, thank you for that — that gives me another angle into this conversation.”’*

(GP10)

*‘And if I don’t think I can do it* [explain information] *, they just have to make another appointment with the specialist and I will call the specialist to say “they have not understood a thing, you have to discuss it again”.’*

(GP07)

### Interprofessional SDM: GPs’ added value

Although they mentioned many examples of practices that support SDM about advanced cancer treatment, most GPs suggested that — when talking about SDM in abstract terms independent of patient cases — they were hardly involved. Cancer treatment decisions were considered mainly the expertise and responsibility of oncologists. Also, GPs reported that patients were primarily hospital-oriented and GPs only acted on patient demand:

*‘Usually, I have no say in this* [treatment decision making] *. I don’t see patients again until after they’ve made a decision with the oncologist … They hardly ever come to me regarding a decision about whether to start chemotherapy. That’s usually beyond my scope.’*

(GP09)

Moreover, some GPs mentioned being cautious about interfering with hospital treatment decision making, reflecting their perception of the role boundaries between oncologists and GPs:

*‘If they’ve even already decided on something with the specialist and started that, then it’s a bit like … well, meddling in a decision that’s already been taken. So you don’t go, uhm, causing trouble.’*

(GP11)

Nevertheless, GPs recognised their potential added value in treatment decision making and mentioned several reasons for this:

#### The unique position of the GP in the healthcare system

GPs pointed out their position as gatekeepers for specialised hospital care. The availability and accessibility of GPs may result in patients contacting them more easily:

*‘Well, I do think that visiting a GP is an easier step than making a new appointment with a medical specialist in hospital. Many questions patients have, take us one or two phone calls to answer or ease their minds, whereas to see a medical specialist they need to make another appointment, another trip to hospital, waiting rooms, and, you name it.’*

(GP05)

GPs believed that their longstanding relationships with patients enabled them to better tailor conversations about decisions than oncologists by accounting for patients’ medical history and social context:

*‘But I also think that a GP is better qualified to check certain motives, more so than a specialist would. Think of certain aspects, like, what will family think of specific decisions? ’*

(GP14)

#### Additional and different conversations about treatment

GPs indicated that their involvement offers patients an additional opportunity to deliberate on their treatment decision, which possibly reduces the sense of urgency and emotional load that may be present shortly after diagnosis. This way, patients have time to let the news settle and think about questions regarding treatment options:

*‘Of course, it’s a very tense conversation, a bad news consultation like that. It often means decisions need to be made at short notice. I think the whole setting itself makes it difficult, where, once patients hear the word “cancer”, they miss out the rest of the conversation. So I think it’s definitely a good idea to have a second conversation about it.’*

(GP07)

#### Primary healthcare provider in the terminal stage

In the Netherlands, GPs become the primary healthcare provider in later stages of palliative care. Some GPs pointed out that because of their specific expertise in this phase, they are able to help patients to anticipate the care offered if they choose to refrain from life-prolonging treatment or when no further life-prolonging treatment options exist:

*‘Then I’ll also discuss my part in that* [terminal phase] *, as in, “what can I do for you? … I can make you as comfortable as possible, that’s my part. So with regard to pain control, chest tightness, nausea, things like that, weight loss, to respond to that as well as possible”. To me, that’s my role as GP, to guide them in this, but definitely also to state very clearly what other options may be, or how I may help at home, outside of hospital.’*

(GP10)

As medical generalists, GPs indicated that they might be less focused on treating the disease than oncologists, thereby providing more space to consider refraining from disease-targeted treatment:

*‘Well, I also explain a little, like … We ask a specialist to do what’s possible, but not everything that’s possible may be beneficial … That is pretty much the specialist’s tunnel vision: we provide treatment. Where we* [GPs] *come in from the angle of: “what is good for you?”’*

(GP04)

Several GPs mentioned that being involved in early decision making about palliative treatment also helped build their relationship with the patient in preparation for the terminal phase:

*‘Really, from the moment of diagnosis I make sure I keep in touch by calling now and then. And over time you see that contact intensifies slightly. And at a certain point, someone’s treatment is exhausted and they’re handed over to me. And I try to not make that moment the first time I see them and have to work up a plan.’*

(GP01)

### Requirements for fulfilling a role in the SDM process

In the interviews, GPs identified some requirements for their involvement in decision making about cancer treatment.

#### Collaboration with the oncologist

Good collaboration with oncologists was considered key for increased and valuable involvement of GPs. Many GPs also indicated that more insight into conversations between the patient and oncologist, and adequate reporting of such conversations, would be helpful:

*‘Yes, I think I’d like to know more about that* [treatment decision making] *process and what is discussed, because you get the idea people get a more positive image than I have … I get the idea people think: “now I’m cured”. While I think: “well yes, you got a stay of execution”.’*

(GP09)

#### Information about cancer and treatment options

GPs believed that limited knowledge of and experience with cancer and cancer treatment restricted their contribution to decision making.

Some GPs described the risk of providing patients with incorrect information. Information provided by oncologists about the diagnosis, treatment, and prognosis was therefore considered helpful:

*‘But I notice, I’m not really trained to know: what chemotherapy, which side effects, life expectancy at which kind of metastatic cancer. But I’d certainly benefit from knowing that.’*

(GP08)

#### Time to engage in conversations about treatment

Several GPs stated the importance of having sufficient time to engage in conversations about treatment. Having enough time would reduce a sense of pressure and help build trust:

*‘It’s a conscious choice to visit someone at 5 pm. And that’s what I tell them: “I’d rather not come around lunch time, because I’ll have to rush and only have 10 minutes or 20 maybe. And this is not an in-between conversation, so I’ll come by around 5 and we can discuss this at length”.’*

(GP03)

#### Trusting relationship with patient

A trusting relationship was described as essential. GPs indicated that having high-quality contact and pre-existing relationships with patients was important to support patients emotionally, comfort them, and build trust. According to some, relationship building helped with discussing patient values and weighing these:

*‘So you can say: “gosh, you’ve had some really bad news. I know you’ve always … You’ve always said I want to turn 100 and how do you feel about that now?”’*

(GP07)

#### Patient-centred communication

Patient-centred communication was considered important. GPs explained they needed skills to adapt conversations to different patient characteristics such as patients’ level of acceptance of their imminent death, health literacy, and spirituality. The ability to set aside personal preconceptions and to converse in a neutral and unprejudiced way were also regarded as necessary, to avoid influencing the patient’s decision-making process:

*‘But I think the most important thing is just no taboos. Being open to discuss everything and really listen. Don’t give your own interpretation of what would I do, if … But really hear what the patient’s fear or need is. I think that’s the most important thing. And then see if you can somehow combine that in such a way that you actually let patients answer that question* [what to do] *themselves.’*

(GP02)

## DISCUSSION

### Summary

GPs, in this study, who are involved with patients with incurable cancer report practices that potentially support SDM: checking the quality of the decision, complementing SDM, and enabling SDM. Even though most GPs believe that decision making about systemic cancer treatment is primarily the oncologist’s responsibility, they do recognise their added value to interprofessional SDM. They refer to their accessibility and longstanding relationships with patients, the additional opportunities they offer patients to discuss treatment decisions, and their expertise as primary healthcare providers in the terminal phase of life. GPs report that requirements for an optimal supporting role in SDM are a good collaboration with oncologists, sufficient information about the disease and its treatment, sufficient time to engage in conversations about treatment, a trusting relationship with patients, and patient-centred communication.

### Strengths and limitations

A qualitative design helped the authors to gain an in-depth understanding of GPs’ experiences. By using an example case as a conversation starter for the interviews, the authors attempted to focus the discussion on the early palliative phase and decisions about disease-targeted treatment. However, using this example case could have unduly directed participants’ responses in parts of the interview. GPs did not know the reason for the patient’s visit or the progress of the decision-making process, which may have caused them to be more hesitant initially when discussing their contribution to SDM. Additionally, even though data saturation was reached, the study might have benefited from the inclusion of a more diverse range of GPs. Most of the GPs who participated in the study were employed in urban areas and indicated having strong affinity with palliative care, which may have affected their views.

### Comparison with existing literature

In line with the findings of this study, other studies have reported that GPs’ involvement in caring for people with advanced cancer is common,^31^^–^34 and is perceived as valuable.^23^^,^^27^^,^^37^ Descriptions of involvement include practices that may support SDM, for example, by clarifying diagnoses and adverse treatment effects, and by acting as an intermediary between patients and medical specialists.^25^^,^^35^^,^^43^ However, many such descriptions were not in the context of SDM nor explicitly identified as supporting SDM. This study adds an in-depth description of Dutch GPs’ perspectives regarding their role in the treatment decision-making process of patients with advanced cancer. Moreover, the findings of the current study identified an additional type of GP involvement: checking the quality of treatment decisions. This seems to be an important intervention to discover patients’ doubts and/or needs, and puts GPs in a monitoring role. The conceptualisation of the GP’s role in SDM helps us to understand how SDM is carried on through relationship-based care and interprofessional collaboration.

GPs described that longstanding relationships with patients enables them to support patients in decision making after a cancer diagnosis. In terms of Haggerty *et al* ’s^26^ categorisation of continuity, GPs outlined the importance of elements of so-called relational continuity (the *‘ongoing therapeutic relationship between a patient and one or more providers’*), informational continuity (the *‘use of information on past events and personal circumstances to make current care appropriate for each individual’*), and management continuity (the *‘consistent and coherent approach to the management of a health condition that is responsive to a patient’s changing needs’*). All types of continuity of care present in family medicine seem to facilitate a supporting role in SDM about cancer treatment.

The results of the current study also show that GPs make an important contribution to interprofessional SDM. Although oncologists have decisional responsibility, GPs may help identify patients’ decisional needs and ensure that these are responded to. Previously, the authors of the current study interviewed hospital nurses about their role in SDM about life-prolonging treatment and extracted similar categorisations of SDM support.^40^ Although their roles are not identical, nurses and GPs might both be regarded as *‘decision coaches’*:^42^ *‘a health professional who is trained to support the patient’s involvement in healthcare decision making but who does not make the decision’*.^22^ The importance of healthcare professionals cooperating to reach high-quality decisions is stressed by Légaré and others,^22^^,^^44^ who proposed an interprofessional model of SDM in which several healthcare professionals, including a decision coach, are involved in the SDM process. Both the findings of the authors’ previous study involving hospital nurses,^40^ and the findings of the current study involving GPs show that healthcare professionals who are already involved in a patient’s care may take on the role of decision coach without the need to involve additional healthcare professionals.

GPs did seem to struggle with interprofessional boundaries: who is responsible for and should be involved in which part of the collaborative SDM process. This may possibly explain some of the requirements they described, such as good collaboration with oncologists and having adequate information about the disease and its treatment. In addition, it may explain the discomfort experienced with ‘meddling’ in decision making, as GPs need to negotiate the tension between ensuring the quality of decisions while at the same time preventing unnecessary doubts and confusion. The importance of collaboration between healthcare professionals was confirmed in a study that evaluated the effect of actively facilitating GP–patient conversations about the treatment decision.^45^ These conversations were often realised only after the decision had already been made in the hospital and, possibly as a consequence, decreased rather than increased patient-perceived SDM.

### Implications for research and practice

There is a growing body of evidence about the GPs’ role in cancer patients’ care and about interprofessional collaboration. In order to adopt an interprofessional model of SDM in advanced cancer care and to help its implementation, future research should explore the perspectives of oncologists, patients, and caregivers about the supporting role of GPs in SDM. To investigate the generalisability of the current study’s findings, it would be valuable to examine whether GP support in SDM would also apply to decisions in non-oncological and non-palliative care settings, as well as in other geographical areas and other healthcare systems, with no universal coverage and/or gatekeeper system.^46^

The proposed conceptualisation of how GPs can support SDM indicates how SDM could be administered through relationship-based care and interprofessional collaboration. To improve this collaboration and facilitate GPs’ involvement, ‘time out conversations’ (TOC), proactively organised conversations between patients and GPs about cancer treatment decisions, show promising results.^30^^,^^45^^,^^47^

Additionally, training GPs effectively in SDM support might increase insight into and awareness of GPs’ contribution to the decision-making process. This may make their involvement more conscious and hence more effective, allowing GPs to safeguard high-quality treatment decisions that are conscious, informed, and appropriate for patients with incurable cancer.

## Data

The authors are not able to make the transcripts of the interviews (publicly) available. Readers can contact the lead author if they are interested in the transcripts.
